# Facilitating psychosexual adjustment for women undergoing pelvic radiotherapy: pilot of a novel patient psycho‐educational resource

**DOI:** 10.1111/hex.12424

**Published:** 2015-11-09

**Authors:** Franchelle Lubotzky, Phyllis Butow, Kathryn Nattress, Caroline Hunt, Susan Carroll, Andrew Comensoli, Shannon Philp, Ilona Juraskova

**Affiliations:** ^1^ School of PsychologyThe University of SydneySydneyNSWAustralia; ^2^ Centre for Medical Psychology and Evidence‐based Decision‐making (CeMPED)University of SydneySydneyNSWAustralia; ^3^ Sydney Cancer CentreGynaecologic Oncology GroupRoyal Prince Alfred HospitalSydneyNSWAustralia; ^4^ Sydney Nursing SchoolThe University of SydneySydneyNSWAustralia; ^5^ Department of Radiation OncologyRoyal Prince Alfred HospitalSydneyNSWAustralia

**Keywords:** anorectal cancer, booklet, dilators, gynaecological cancer, qualitative study, radiotherapy, sexual adjustment

## Abstract

**Purpose:**

This pilot study aimed to obtain feedback on the feasibility, safety and acceptability of a psychosexual rehabilitation booklet developed for women undergoing pelvic radiation therapy (PRT) and to explore women's sexual, informational and supportive care needs post‐PRT rehabilitation.

**Methods:**

Twenty women treated with PRT for gynaecological or anorectal cancer within the last 5 years, who had received vaginal dilators, provided feedback on the format, content and utility of the booklet and discussed their post‐treatment information needs, via a semi‐structured phone interview. Women completed standardized (HADS, IES‐R) and study‐specific scales to characterize psychological status of the sample and to assess participants' booklet knowledge and feedback, respectively.

**Results:**

The booklet was perceived as very helpful, informative and not distressing, providing additional information to that discussed with clinicians. After reading the booklet, women had good understanding of strategies to reduce the sexual impact of PRT. Many women reported that discussion of sexuality was often avoided during consultations, despite them experiencing distressing sexual experiences and difficulties post‐PRT.

**Conclusions:**

This novel resource which addresses an important component of post‐pelvic radiation care appears acceptable and highly valued. Findings have highlighted a need for sexual health communication training for clinicians who treat this population so that they can initiate conversations about vaginal health and sexual health in an informed and comfortable manner. The impact of the revised booklet on psychosexual and clinical outcomes is being evaluated in a multicentre RCT.

## Introduction

A common adjuvant treatment for women with gynaecological and anorectal cancer is pelvic radiation therapy (PRT). Both external beam PRT and vault brachytherapy can result in debilitating acute and late effects, including physical side‐effects affecting the bowel (obstruction, stricture, fistulae and perforation), urinary system (haematuria, urethral stenosis and vesicovaginal fistulae) and vagina (dryness, fibrosis, stenosis and agglutination).^1^, ^2^ Approximately half of gynaecological cancer patients report on‐going sexual morbidity following PRT,^1^, ^3^, ^4^ with women reporting this as a major cause of distress to them^5^ with this morbidity commonly accompanied by depression and anxiety^6^ and poor quality of life.^7^ In female rectal patients #bib62% report sexual dysfunction and morbidity post‐treatment, with preoperative radiotherapy representing the only significant risk factor.^8^ Overall, there is a paucity of studies and underreporting of sexual side‐effects, particularly in women with anorectal cancer.^9^ Thus, both gynaecological and female anorectal patients report chronic and distressing post‐treatment physical and psychosexual side‐effects, which persist long after other areas of life have returned to normal.^10^, ^11^, ^12^ Despite this, female sexual morbidity post‐PRT remains a neglected aspect of follow‐up clinical care.^11^, ^12^

Vaginal stenosis, in particular, is a well‐recognized pelvic radiation complication in women receiving PRT.^13^, ^14^ After radiation treatment to the pelvic area, scar tissue begins to form in the vagina with the tissue becoming less elastic and dry, resulting in adhesion formations. Adhesions are bands of scarlike tissue that form between two surfaces inside the body and cause them to stick together. Over time as a part of the healing process, the body will either break down the adhesion and replace it with normal tissue, or form a permanent adhesion.^15^, ^16^ The use of vaginal dilators is recommended to prevent adhesions progression to fibrosis and stenosis of the vagina, especially during the first year after completion of radiotherapy in order to maintain vaginal patency (UK guidelines). If the adhesions are not broken down on a regular basis, contractions, shortening, narrowing and for some women complete obliteration of the vagina may develop.^15^

There is some evidence that vaginal stenosis can be minimized or prevented with appropriate interventions such as the use of vaginal dilators.^14^, ^17^, ^18^ Compliance with dilator use has been associated with increasing vaginal comfort and control of pelvic floor muscles,^20^ and preserving overall vaginal health and sexual function.^17^, ^18^, ^21^, ^22^ Dilator use compliance also enables adequate pelvic examinations to monitor for any recurring changes in vaginal tissue, an important element of cancer surveillance.^17^, ^18^, ^20^, ^22^ However, there remains a lack of good‐quality evidence for the use of vaginal dilators.^19^ This may simply reflect inadequate evidence base due to a lack of prospective, controlled, longitudinal studies explicitly exploring the efficacy and safety of vaginal dilators.^19^ Despite the aforementioned issues, the use of vaginal dilators is recommended as standard practice once the acute inflammatory phase has settled (i.e. about 6 weeks post‐PRT)^17^ by The American Cancer Society^23^ and The UK National Forum of Gynaecological Oncology Nurses.^24^

The lack of validated resources for sexual rehabilitation post‐cancer treatment and cost‐effective ways of providing them is a significant problem,^22^ considering over 80% of women with gynaecological cancer report wanting detailed information about their disease, its treatment and self‐care strategies.^25^, ^26^ Tailored information provision about potential side‐effects and effective self‐care in gynaecological cancer has been linked to better coping with side‐effects, better compliance with post‐radiation rehabilitation, less fear about sexual intercourse and less relationship disruption.^25^

Despite these benefits, the provision of dilators and dilator use information is often inadequate, and practices currently vary within and between treatment centres, resulting in suboptimal compliance with this potentially beneficial rehabilitation aid.^25^, ^27^ Commonly cited barriers to dilator use include uncertainty regarding how/when to use dilators, viewing it as a negative experience^28^ or ‘embarrassing sex toy’ [29, p. 1162] and ‘reliving the invasion of treatment’ [29, p. 1162]. Additional practical barriers to dilator use and sexual rehabilitation also arise in cases when sexual or pelvic floor rehabilitation physical therapists are not readily available or when not clear the referral pathways are established. This is of concern, as women would benefit from access to these services to obtain effective guidance with vaginal (scar tissue) care and general sexual recovery after radiation treatment.^20^, ^30^ By contrast, factors facilitating dilators use include concern about stenosis, belief that dilators work, reminders of stenosis, and acceptance of dilator use as part of the woman's normal routine or an extension of medical treatment.^28^

Doctors are often uncomfortable discussing post‐treatment sexual adjustment, and may incorrectly assume that women have a comprehensive understanding of female reproductive anatomy.^25^ Discussing sexual adjustment can also be difficult with sexual concerns often unvoiced by patients,^14^ particularly by older women, couples and those with cultural or religious constraints.^25^, ^31^ Overall, it is likely that patients and health‐care professionals would benefit from resources to facilitate information provision regarding sexual adjustment, potentially increasing uptake of interventions with clinical import and improving quality of life for gynaecological and anorectal cancer survivors.

The current study aimed to investigate the feasibility, safety and acceptability of providing women undergoing PRT for gynaecological or anorectal cancer with a psycho‐educational booklet, to improve information delivery about:

1. Radiation‐induced side‐effects potentially affecting post‐treatment sexual functioning, and
2. Rehabilitation options and evidence‐based self‐care strategies, such as the use of vaginal dilators, to prevent/minimize vaginal changes.

Women's views about rehabilitation infor‐mational needs following PRT and post‐treatment sexual adjustment were also explored.

## Methods and materials

### Participants

Women treated for definitive or adjuvant PRT (i.e. not palliative treatment) at a tertiary cancer centre were approached to participate in the study. Eligible women were identified by clinicians either from the clinic database or at their follow‐up clinic visit and invited to participate via post or in person. Eligibility criteria stipulated that women: had undergone PRT for gynaecological cancer or anorectal cancer; were less than 5 years post‐treatment; had been given dilators; were 18 years or more of age; were English speaking; and had no concurrent psychiatric or cognitive disability (screened by recruiters/clinicians). Ethics approval for the study was obtained from the relevant area health service human research ethics committee.

### Booklet

The content of the PRT booklet was based on a comprehensive literature review and existing general information resources, for example, Australian Cancer Council booklets on radiation/sexuality and cancer/women's pelvic region cancers.

Standardized principles for the development of patient resources, namely C.R.E.D.I.B.L.E. Criteria,^32^ were used to guide the booklet development. Drafts of the booklet were reviewed using an iterative process involving discussion between the research team, three consumer representatives and health professionals working in this area at three major tertiary hospitals, including doctors (radiation oncologists and gynaecological oncologists), specialist cancer nurses, a physiotherapist and a clinical psychologist. The final booklet content is presented in Table 1. A copy of the booklet can be downloaded from the following NSW Cancer Institute radiation oncology (colorectal and gynaecological cancer) webpage: https://www.eviq.org.au/Home.aspx.

**Table 1:** Content of the information booklet

Content of the information booklet
Internal and external pelvic radiation therapy
Pelvic radiation therapy side‐effects (includes diagrams of female reproductive organs)
Sexuality during and after pelvic radiation therapy (e.g. pain during intercourse), dispels myths about treatment and sexuality
Coping with sexual difficulties after radiation
Practical strategies to maximize vaginal health after radiation treatment – vaginal dilators, lubricators, pelvic floor muscle (PFM) exercises
Psychological/emotional effects of pelvic radiation therapy
Where you can get information, support contacts and resources
References and glossary of terms
Asking questions can help: lists prompt questions to ask health professionals
Useful health‐care team contacts: section to list name, number and email
My recovery diary: lubricant use, sexual activity, dilator use, any difficulties

### Procedure

Each participant was provided with the study pack containing an invitation letter from a doctor/nurse and the researchers, a consent form, an information sheet, the booklet, a study questionnaire and a reply‐paid envelope. Completed questionnaires and signed consent forms were returned within 2 weeks of receipt. Following this #bib45‐ to 60‐min semi‐structured telephone interview was conducted with each participant to obtain feedback about the booklet format, content, clarity, usefulness, section relevance and improvement as well as vaginal changes, sexual adjustment and informational needs post‐PRT.

### Measures

The study questionnaires included the following standardized measures (HADS, IES‐R), employed to characterize the psychological status of the sample. Two additional scales, specifically designed for the study, assessed participants' understanding (Knowledge scale) and acceptability of the booklet content and format (Feedback scale), to inform the next stage involving a randomized controlled trial of the booklet.

*The psychological status of the sample* was assessed using the validated 14‐item *Hospital Anxiety and Depression Scale (HADS)* 33 measuring anxiety (seven items) and depression (seven items) among physically ill patients. High scores reflect high levels of anxiety or depression symptoms.

*Levels of subjective distress/post‐traumatic stress* were assessed using the *Impact of Event Scale‐Revised (IES‐R)*.^34^ The *IES‐R* has 22 items measuring intrusion, avoidance and hyperarousal. High scores reflect high levels of post‐traumatic stress symptoms.

The 15‐item *Knowledge Scale,* specifically designed for the study, assessed women's knowledge and understanding of the booklet content (e.g. post‐PRT sexual side‐effects, potential rehabilitation options). Response options were true/false or ‘don't know’. A total score (0–5) is calculated by summing items, with high scores reflecting good understanding of the booklet content.

The 19‐item *Feedback Scale,* also specifically designed for the study, was used to elicit views on the format and content of the booklet (e.g. confidence in using and awareness of the potential importance of using rehabilitation strategies). The Feedback scale was based on a similar scale used in previous pilot study evaluating an acceptability of a communication resource, namely a decision aid.^35^ Response options were on a 5‐point Likert scale, ranging from ‘Strongly Disagree’ to ‘Strongly agree’ with a ‘Not sure' option. High scores reflect positive feedback on the format, content and utility of the booklet.

*Demographic and clinical information* was also collected, including age, relationship status, education, occupation (see Table 2).

**Table 2:** Demographic and clinical characteristics, and psychological status of the sample (*N* = 20)

Variable	Value
Age	
Mean	55.20
SD	11.92
Min–max	38–82
Age at diagnosis	
Mean	52.65
SD	11.89
Min–max	37–81
Relationship status n (%)	
Single	4 (20%)
Married/de facto	11 (55%)
Separated/divorced	2 (10%)
Widowed	3 (15%)
Education	
High school	3 (15%)
TAFE certificate, diploma, business college	4 (20%)
University degree	13 (65%)
Presently working	
Yes	14 (70%)
No	6 (30%)
Type of cancer	
Gynaecological	14 (70%)
Anorectal	6 (30%)
Type of treatment	
Surgery, radiotherapy and chemotherapy	6 (30%)
Surgery and radiotherapy	5 (25%)
Radiotherapy and chemotherapy	8 (40%)
Radiotherapy only	1 (5%)

	Psychological status	
	Mean	SD
Scale		
HADS anxiety	5.30	4.40
HADS depression	3.55	2.87
IES‐R avoidance	0.89	0.85
IES‐R intrusions	0.79	0.71
IES‐R hyperarousal	0.57	0.64
IES‐R total	0.75	0.63

HADS, Hospital Anxiety and Depression Scale; IES‐R, Impact of Events Scale‐Revised.

### Data analysis

A standard content analysis, a method of categorizing information based on frequency of occurrence, was employed to analyse the interview data.^36^ The initial coding scheme was developed via an iterative process involving a panel of expert researchers (FL, IJ, KN, PB). The coding scheme included clear definitions and examples for each key theme identified. A proportion of the data set was double coded by two researchers with differences resolved by discussion. Frequencies of response occurrences for each theme were counted. Sampling continued until no new themes emerged in three consecutive interviews (i.e. theoretical saturation),^37^ and this was achieved with 20 respondents.

## Results

Of the 66 women invited to participate #bib20 (30%) responded. The panel of experts deemed that a theoretical saturation was reached with the current sample as no new themes were emerging.^37^ Therefore, no further invitations were required to be sent out to participants. The final sample characteristics are presented in Table 2. The average age at the time of the study was 55 years, just over half (55%) were married and almost half (45%) had a university education.

### Psychological characteristics

On the HADS, most women (*n* = 16) fell within the normal range (0–7 out of 21). Two women scored in the subclinical (8–10 out of 21) and two in the clinical (11–21) range for anxiety and two women fell in the subclinical range for depression (8–10 out of 21). On the IES‐R, again most women reported normal symptom levels (*n* = 18, where normal = 0–8.5 out of 88), and two women reported clinical (>33 where high symptom levels = 19–88) levels of post‐traumatic stress symptoms.^38^

### Knowledge/Understanding of the booklet content

All women answered 8 of 15 or more items correctly on the *Knowledge Scale*, with a median score of 13/15, indicating good overall understanding of the booklet content (see Table 3). The least understood item regarded vaginal dilator care, with only 12 patients answering this item correctly.

**Table 3:** Knowledge Scale: women's knowledge of information contained in the booklet (*N* = 20)

Items	Correct (*n*)	% Correct
The term vaginal stenosis means the narrowing of the vaginal walls (T)	18	90
The use of vaginal dilators is recommended 3–5 times per week (T)	19	95
Dilators need to be used for 5–10 min at a time (T)	18	90
Dilators need to be used for no longer than for 1 year after your treatment (F)	15	75
Using vaginal dilators can help in making sexual intercourse less painful (T)	19	95
It is not important to use vaginal dilators for pelvic examinations by your oncologists (F)	17	85
Cancer treatment can affect your emotions (T)	18	90
All women receiving pelvic radiation therapy will experience sexual difficulties (F)	17	85
There are people to talk to about your sexual life if you need help after your treatment (T)	19	95
Sexual intercourse can spread cancer (F)	20	100
Pelvic radiation therapy can make you ‘radioactive’. Therefore, it is important to be careful that this is not transferred to your partner (F)	20	100
All vaginal changes from pelvic radiation therapy will happen in the first few months after treatment (F)	13	65
It is advised that you use lubricant when using the dilator (T)	20	100
Dilators require special procedures to keep them hygienically sterilized after each use (F)	12	60

Note: T = true; F = false

### The format and utility of the booklet: quantitative results

All of the women (100%) found the booklet format easy to read and understand. The booklet was found to be helpful (85%) and useful (80%) by the majority of women (Table 4).

**Table 4:** Feedback Scale: women's quantitative feedback on format and content of the booklet (*N* = 20)

Item	Strongly disagree/disagree *n*	Neutral *n*	Strongly agree/agree *n*
Easy to read	0	0	20
Easy to understand	0	0	20
Information was confusing	19	0	1
Booklet made me anxious	17	0	3
Too long	19	0	1
Too detailed	19	0	1
I liked the overall format	1	1	18
I know which options are available to me	1	0	19
I have acquired new information	1	0	19
After reading the booklet, I feel confident using vaginal dilators	2	0	17
Booklet provided additional information to that provided so far	3	0	17
I felt uncomfortable with the personal nature of the information	16	2	2
Booklet was helpful	2	0	18
Booklet gave me information I needed	2	2	16
Booklet gave me information I did not have	4	1	14
I know why it is important to use vaginal dilators	1	0	19
I found it easy to find the information I wanted	1	2	16
I like the way the booklet looked	2	1	17

### Booklet feedback: qualitative results

Women endorsed the importance of receiving information about post‐treatment sexual adjustment and gave strongly positive feedback about the content, format and utility of the booklet. In particular, the booklet was valued for breaking down the sexuality taboo. Specific feedback was categorized into the following themes: (i) experiences of silence regarding sexual health in care and need for information resources, (ii) booklet validating sexuality, (iii) booklet useful throughout the cancer journey, and (iv) booklet rehabilitation strategies acceptability.

#### Experiences of silence regarding sexual health in care and need for information resources

Women emphasized experiencing distressing vaginal changes and sexual difficulties following PRT, and the importance sex had in their lives. Some felt they were failing their partner. Despite this, many women reported their experience of care and the provision of information concerning sexuality and the use of dilators had not been optimal. They had a perception that their health‐care team were uncomfortable raising issues about sexuality, describing it as something hidden, avoided and ignored.

I couldn't talk about sexual issues, felt with the (male) doctor this is woman's stuff – talk to a woman was the prevailing idea. Vaginal changes did not enter into these conversations. I had a feeling of failure, shortcomings, that I was not delivering for my partner. (ID D109)

Many were unsure as to whether it was their doctors' role to discuss sexual matters and vaginal changes.

…due to some physical and emotional circumstances, my sexual activity since surgery/treatment is not the same …I find this very regrettable. My doctor was fantastic but I got the general gist after radiotherapy that the less is said (about sexual issues) the better … It was frustrating. It's important to validate sexuality issues and to know I'm not alone. (ID D109)

…the dilator situation is all very much hidden and not discussed much, sometimes male doctors do not like to discuss much… (ID D112)

Women expressed a strong need for resources such as the study booklet to validate and to overcome their difficulties with sexual and vaginal changes. The majority of anorectal cancer patients reported being comforted by having a booklet specific to them, which addressed sexuality, with additional significance for those with anal cancer, reflecting the stigma they could feel about these issues.

I felt that I had little support on my journey with anal cancer, there was no booklet for me, I was so relieved to see this – it was so comforting and empowering that my sexual life was being addressed…. You're so desperate, because with anal cancer you don't fit into other categories and so much is linked with the HPV virus. Both anal cancer and talking about sexual issues have seemed like taboo subjects. (ID C031)

Even before opening the booklet it was great, just the fact that there is a booklet on this. I felt informed and comfortable that the information was there. (ID D109)

#### Booklet validating sexuality

Many women found the booklet validated their sexual concerns and felt that information on sexual issues was highly beneficial.

(The study booklet was) spot on, I felt jubilant finally, thank you, some validation, confirmation, put in writing, I felt so alone, I realised what didn't correspond, my sexual activity has never gone back to what it was before. (ID D109)

Woman highlighted how much the booklet reduced their distress and alienation, facilitating and giving confidence to addressing their sexuality by bringing these topics into the open. Knowing what to expect reduced fear, and the information provided was found empowering.

I thought to myself I am so glad I've got this (booklet) because I've just been given the dilators and (that) was a struggle in itself. It was a good bible to use, I referred back often, I was glad I had it… (it) reassured me. (ID C009)

I was very impressed with the booklet, it was something new and fresh and it didn't instil any fear… the best thing I could have done was to read this booklet. (ID D110)

…the booklet gave me confidence to resume sexual activity. Giving me information helped me to take control, confidence to go for it. Information is power – let's give it a go. It made me feel confident to have sex. (ID C031)

…If you follow (information in the booklet) it will help you afterwards. I felt conveyer‐belted – had a sense of powerlessness – knowledge is power – gives you more control in the process. It ensures better control of your health long‐term. (ID C028)

Women were asked to comment on specific sections of the booklet, with most finding the female anatomy diagrams and sexual side‐effects information acceptable and helpful as educational tools, validating their experience and providing permission to discuss their needs.

…it's preferable to see it here (in the booklet) where's in consultation with your doctor a patient might feel embarrassed showing ignorance. (ID ROC10)

I thought it was all important because there are those consequences, not only physical. Having it (the sexual side effects) written down validates feelings and encourages you to talk (to the health care team) if having problems. (ID C035)

#### Booklet useful throughout the cancer journey

Most women strongly felt that they would have liked to have had the booklet at the time of their treatment to address any concerns they had.

…I thought (the booklet) was great; though wish I'd had it at the beginning of the (treatment) process. I was concerned early on about the effects of radiation, narrowing and tightening and menopause… My sex life was an important consideration for me. (ID C033)

Furthermore, the booklet was helpful even after an extended period post‐treatment which is in line with research and clinical evidence that side‐effects can present years post‐treatment.

I have recovered (from gynaecological cancer) about 5 years now and I still found some of the information useful. (ID D112)

In addition, the resource was found useful across different age ranges, varying needs and for partners and family.

I'm 82 so all does not apply but does in a way. I found it very good… it was not embarrassing.… It was for all ages, thought excellent really. (ID C026)

The (booklet) acknowledged what can happen to younger women. (ID D114)

The booklet wasn't only good for me but it was good for my partner and children, my husband tried to push the effects aside… the booklet can alleviate fear, having knowledge was very helpful. (ID D109)

#### Booklet rehabilitation strategies acceptability

Another important section and main focus of the resource was on ‘Rehabilitation strategies’ (e.g. the use of dilators, lubricants). Women noted the profound distress they experienced with treatment‐related vaginal changes. They felt the booklet provided helpful options on how dilator use may improve their rehabilitation and persevering with this during difficult phases post‐treatment.

…I used the words of the booklet like a mantra after I was struggling with the narrowing of my vagina after my treatment for rectal cancer, I persevered with the dilators and I succeeded, I was so grateful to have the booklet. (ID C031)

If this booklet had been available to me at the time of treatment I may have reduced the scarring in my vagina and not have so much discomfort when having pelvic vaginal examinations done. (ID ROC024)

I thought that the stuff on (vaginal) scar tissue was useful. I hadn't had that described to me in detail before. I found it upsetting that it hadn't been explained and that it could be a permanent problem. This was probably the most useful part of the whole book for me. It would have been good to know this from the beginning. (ID Q047)

…sexually during and after (your) vagina becomes less flexible at the size of it, you really do feel a difference, I felt so traumatised by invasiveness of the brachytherapy, the booklet shows how dilators are important for optimal vaginal health. (ID C009)

I found the information on the brands of lubricants was really helpful …it was helpful to include some information about how to keep dilators clean, where to store them to keep them dry. (ID D104)

Some suggested improvements incorporated into a revised booklet were clarifying that this is a psychosexual resource in response, to, for instance, this comment:

I felt somewhat alienated by the dominance of sexuality – uterine cancer and good health is a woman's primary concern I feel. (ID ROC10)

The revised booklet clarified the content relevance to those not in a sexual relationship, due to the clinical importance of maintaining a healthy vagina, although most women indicated that the booklet conveyed this.

The importance of using vaginal dilators …I was not really aware of this because I'm not in a sexual relationship. You really need to look after your vaginal health for yourself. (ID C042)

## Discussion

This study piloted a psychosexual rehabilitation information booklet for women post‐pelvic radiation therapy (PRT) for gynaecological or anorectal cancer. The pilot revealed no safety issues, and the booklet was found to be acceptable and helpful. Evidence of the booklet's feasibility came from its successful delivery to patients targeted by the booklet during the pilot. The booklet was revised based on participants' interview and quantitative (Knowledge and Feedback scales) responses. The need for this specific psycho‐educational resource was strongly expressed by the majority of women in the study. Importantly, the booklet was found to be helpful even after many years post‐treatment, which is in line with research and clinical evidence that PRT side‐effects and unmet psychosexual needs are evident years following treatment.^10^, ^11^, ^12^

The qualitative findings revealed many unmet needs for women with regard to the physical and psychosexual effects post‐PRT. Women emphasized distressing vaginal changes and sexual difficulties following treatment, and the importance sex had in their lives. Some felt they were failing their partner. Importantly, many women felt their sexual concerns and need for sexual side‐effects information were not validated by the health‐care team, and that generally, sex was a hidden and avoided topic.

These findings are consistent with other studies where many health‐care professionals reported not discussing sexual function with gynaecological cancer patients, citing a lack of knowledge, embarrassment and insufficient resources to provide support as the main barriers.^26^, ^39^ Likewise #bib50% of surveyed gynaecological cancer patients reported receiving little or no information on sexuality and cancer, while 60% wanted more information.^26^ In a similar vein, the NSW Cancer Institute^40^ in Australia found the two largest areas of failure of care for outpatients are ‘not receiving enough information about possible changes in sexual activity' and ‘not receiving enough information about possible changes in relationship with spouse or partner’.

Many women in the current study wanted information about sexual difficulties, to discuss their concerns, play a role and have a choice in their long‐term recovery and health, but found it difficult to raise sexual changes with their doctors, out of not knowing if it was their role. These findings mirrored those of others for both gynaecological and anorectal cancer patients.^22^, ^25^, ^26^, ^27^, ^39^ Women felt that the study booklet would facilitate communication about these issues. Furthermore, knowing about the clinical importance of using dilators, which may enable adequate pelvic examinations to detect recurrence, was helpful in overcoming barriers persevering with using dilators, whether sexually active or not.

Many women wished they had known about the vaginal changes/sexual side‐effects and potential rehabilitation strategies at the time of their treatment, feeling it may have reduced their post‐treatment vaginal changes and sexual difficulties. They felt their fears may have been reduced or eliminated, as opposed to suffering in silence and distress – believing it was important to break the ‘sounds of silence…’ [26, p. 238]. This suggests that the booklet should ideally be made available to women before, or early on in their treatment so that they can anticipate and, with their partners, better prepare themselves psychologically for post‐treatment rehabilitation. The booklet was well received and not reported to be distressing by the majority of women. Anecdotally, the health‐care professionals whom we consulted and those who participated in the pilot expressed a strong desire for the booklet to become available for their patients.

### Limitations and future research

The low response rate for this study reinforces that sexual issues, vaginal changes and dilator use are difficult to discuss.^28^, ^29^ Another contributing factor may have been the time since treatment (up to 5 years) given that this was a retrospective study. Women with more conservative sexual attitudes and those who did not experience adverse side‐effects may have been less likely to participate. Conversely, it is likely that only those participants interested in and comfortable in discussing their sexuality/sexual health took part in the study, which is a potential source of bias. It is also possible that the present study findings do not reflect the views of women who had received only minimal prior clinical communication about sexuality, given that only women who had been given a dilator were included. Finally, participants were all recruited from the same treatment centre, were English speaking and generally well‐educated; thus, these findings may not generalize to other treatment settings with different patient populations.

The use of vaginal dilators as rehabilitation aids post‐PRT is deemed unsuitable by some clinicians and in certain regions of the world. This stems in part from insufficient good‐quality evidence on the efficacy of vaginal dilation in preventing/minimizing PRT‐related late effects or quality‐of‐life outcomes,^41^, ^42^ as well as suggestion that routine dilation during or immediately after PRT may increase the risk of genital tract fistula,^17^ a rare complication which may occur even when dilators are not used.^41^, ^42^ Thus, further research is needed to provide higher level evidence for dilator use in addressing PRT‐related psychosexual and physical side‐effects.^14^, ^17^, ^27^ In addition, identifying the optimal time, context and strategy to convey sensitive information is still a challenge within supportive care in PRT practice.^21^ This has been an area identified for further research, so too has the relationship between written materials and clinical discussions.^22^, ^25^, ^26^, ^27^, ^38^, ^42^

## Conclusion

This study addresses a previously neglected but important component of post‐treatment care for women undergoing PRT for gynaecological and anorectal cancer by obtaining feedback on a novel and much‐needed psychosexual recovery/rehabilitation information booklet. Findings have highlighted a need for sexual health communication training for clinicians who treat this population so that they can initiate conversations about vaginal and sexual health in an informed and comfortable manner. An online evidence‐based, interactive communication skills module has been recently developed in Australia,^43^ with the aim to improve health professionals' skills and confidence in providing effective psychosexual care to women affected by gynaecological cancer, and their partners (see http://modules.cancerlearning.gov.au/psgc/). The current findings also underscore the need for further interventions to address the emotional aspects of treatment and its potential impact on intimate relationships for these women.

Overall, the current study provides strong support for the provision of this psychosexual resource to better support women's rehabilitation post‐PRT. The booklet has been revised based on the feedback from study participants and also current Cochrane review findings.^19^ Given the high levels of acceptability of the booklet, its effectiveness is currently being evaluated in a randomized controlled trial. If shown to be effective, this relatively simple psycho‐educational intervention is potentially transferable to a range of PRT treatment settings.

## Conflict of interest

The authors have no conflict of interest to declare.
